# Occurrence of Arrhythmias in Women with Thyroid Cancer Receiving Suppressive Doses of Levothyroxine

**DOI:** 10.3390/curroncol28060420

**Published:** 2021-11-29

**Authors:** Karol Kaziród-Wolski, Aldona Kowalska, Janusz Sielski, Magdalena Biskup-Frużyńska, Grzegorz Piotrowski

**Affiliations:** 1 Collegium Medicum, Jan Kochanowski University in Kielce, 25-317 Kielce, Poland; aldonako@onkol.kielce.pl (A.K.); JSIELSKI7@interia.pl (J.S.); 2 Intensive Cardiac Care Unit, Swietokrzyskie Cardiology Center, 25-736 Kielce, Poland; 3 Department of Endocrinology, Holy Cross Cancer Center, 25-734 Kielce, Poland; 4 Maria Skłodowska-Curie National Research Institute of Oncology, Gliwice Branch, 44-102 Gliwice, Poland; magdalena.biskup-fruzynska@io.gliwice.pl; 5 Department of Cardiooncology, Medical University of Lodz, 90-549 Łódź, Poland; gpiotr4@wp.pl; 6 Department of Cardiology, Copernicus Hospital, 93-513 Łódź, Poland

**Keywords:** thyroid cancer, arrhythmias, levothyroxine

## Abstract

*Aim of the study*: To assess the occurrence of cardiac arrhythmias caused by high doses of levothyroxine in patients with thyroid cancer with subclinical hyperthyroidism. *Materials and Methods*: This prospective study included 98 women divided into three groups according to plasma thyroid stimulating hormone (TSH) concentration: <0.1 µU/mL, 0.1–0.39 µU/mL, or 0.4–4.0 µU/mL (control group). All participants underwent laboratory tests and an electrocardiography (ECG) Holter test to assess their heart rate and the occurrence of arrhythmias. Statistical analysis assessed differences between groups in all clinical parameters and factors influencing the occurrence of arrhythmias. *Results*: There were no differences between groups in the maximum, average, or minimum heart rate or in the incidence of the studied cardiac arrhythmias. Heart rate in women with a TSH concentration of <0.1 µU/mL depended on age and the presence of arterial hypertension, and heart rate in women with a TSH concentration of 0.4–4.0 µU/mL depended on free triiodothyronine concentration and the presence of arterial hypertension; no relationship was identified for women with a TSH concentration of 0.1–0.39 µU/mL. One-way logistic regression analysis did not identify any factors influencing the occurrence of arrhythmias. *Conclusions*: While maintaining normal free triiodothyronine levels, the use of suppressive doses of levothyroxine after thyroidectomy for differentiated thyroid cancer does not induce clinically significant arrhythmias or affect average heart rate. None of the studied clinical parameters influenced the risk of arrhythmia.

## 1. Introduction

Thyroid cancer is a malignant neoplasm originating from the thyroid epithelial tissue and is the most common neoplasm of the endocrine glands [1]. Papillary cancer is the most common malignant neoplasm of the thyroid gland, accounting for 60–95% of all malignant thyroid neoplasms, with a peak incidence between the ages of 20 and 40 years [2]. Follicular carcinoma accounts for 10–30% of malignant thyroid neoplasms, with incidence increasing significantly after the age of 50 years [3]. Thyroid cancer is almost five times more common in women than in men, and the incidence has increased in recent years [4]. The principles of diagnosis and treatment of thyroid cancer in force during the study are included in the recommendations prepared by the Polish Group for Endocrine Carcinomas in 2010 [5]. The 2015 guidelines of the American Thyroid Association for the treatment of nodules and differentiated thyroid cancer in adults, which are also valid in Poland, were published in January 2016 [6]. Treatment is a multi-stage process and is highly effective. It consists of thyroidectomy, ^131^I radioiodine therapy, and chronic thyroxine therapy, usually with the aim of full or partial thyroid-stimulating hormone (TSH) suppression in order to minimize the risk of recurrence [7]. Full suppression means achieving a TSH concentration <0.1 µU/mL without causing symptoms of hyperthyroidism, while partial suppression means achieving a TSH concentration in the range of 0.1–0.4 µU/mL also without symptoms of hyperthyroidism). Hyperthyroidism is excluded by detection of normal free triiodothyronine levels in the blood. The determination of free thyroxine concentration is not recommended in this situation [5]. Full or partial TSH suppression is associated with the induction of exogenous subclinical hyperthyroidism. So far, a relationship has been demonstrated between hyperthyroidism and the occurrence of arrhythmias such as sinus tachycardia, atrial fibrillation, and supraventricular premature beats [8]. Numerous supraventricular premature beats, apart from having a significant impact on quality of life, are also a significant predictor of atrial fibrillation [9]. Previous studies have shown that endogenous subclinical hyperthyroidism can cause an increased number of supraventricular premature beats; however, the difference compared to the healthy population was not statistically significant [10]. Atrial fibrillation is estimated to occur in 10–15% of patients with hyperthyroidism [11]. The incidence of atrial fibrillation increases with age, and a low TSH concentration is an independent risk factor for this arrhythmia [12].

## 2. Materials and Methods

A total of 98 women were included in this prospective study. Participants were recruited at the Department of Endocrinology of the Holycross Cancer Center in the years 2014–16. The control group consisted of healthy volunteers. The study population was divided into three groups: patients with full TSH suppression (blood TSH <0.1 µU/mL, without causing hyperthyroidism; *n* = 48); patients with partial TSH suppression (blood TSH 0.1–0.4 µU/mL, without causing symptoms of hyperthyroidism; *n* = 25); and a control group of healthy women (*n* = 25).

In patients with thyroid cancer, the determination of TSH concentration, and the dose of levothyroxine were adjusted every 6 months. The following rules were adhered to: replacement doses were used in low-risk patients, relative suppression was maintained in the case of intermediate-risk, and full suppression was maintained in high-risk patients. Presented TSH concentrations was a one-off measurement performed just before the Holter electrocardiography (ECG) test.

All study participants gave their written consent to participate in the study. Ethical approval was obtained from the Bioethical Committee at the Jan Kochanowski University in Kielce (No. 3/2014).

Included individuals did not use antiarrhythmic drugs and had not been diagnosed with cardiovascular diseases such as heart failure, ischemic heart disease, myocardial infarction, a congenital or acquired heart defect, or arrhythmias (supraventricular or ventricular arrhythmias). Patients with the above-mentioned conditions were excluded on the basis of an interview and available medical documentation. Eligibility for the study was based on the exclusion of disqualifying symptoms on physical examination—i.e., loud systolic murmur (at least 3/6 on the Levin scale), diastolic murmur, and symptoms of heart failure (such as dyspnea or features of fluid retention on physical examination).

All participants in the study underwent a 24-h Holter ECG test with a 3-channel AsPEKT 702 apparatus (ASPEL, Zabierzów, Poland) equipped with a memory card and a battery power source. The recorded data were analyzed by the authors of the study at the ASPEL Holcard 24w computer data analysis stand. Eligible participants had an ECG recording lasting at least 21 h and a maximum of 5% of artifacts. The occurrence of arrhythmias such as supraventricular and ventricular extrasystoles, paroxysmal supraventricular tachycardias, and atrial fibrillation was assessed. The quantitative norms for these arrhythmias are presented in Table 1.

Part of the patients was diagnosed with hypertension before enrollment. The diagnosis was made based on the following values of blood pressure: ≥140 mmHg (systolic) and/or ≥90 mmHg (diastolic) in the doctor’s office; ≥135 mmHg and/or ≥85 mmHg in-home measurements. The diagnosis was also made based on outpatient blood pressure monitoring (ABPM), when the diagnosis was made when the measurements were ≥135 mmHg and/or ≥85 mmHg during the day, ≥120 and/or ≥70 mmHg during the night or ≥140 mmHg and/or ≥90 mmHg as average values over the whole day.

Descriptive statistics are presented for quantitative parameters, including the arithmetic mean with standard deviation for variables with a normal distribution and the median with interquartile range for variables without a normal distribution. For qualitative parameters, numbers and percentages are presented. Both parametric and non-parametric tests were used to assess the statistical significance of differences within the studied groups. For quantitative variables, parametric tests (*t*-test for two groups and one-way ANOVA) were used when data were normally distributed, and homogeneity of variance was maintained. The nature of the distribution was assessed by the Shapiro-Wilk and Kołmogorov-Smirnov tests. Equality of variances was assessed with Levene’s test. If the required assumptions were not met, the Wilcoxon test was used for two groups and the Kruskal-Wallis test was used for more than two groups. For qualitative parameters, crosstabs and the Chi-square test were used. When statistically significant differences were found, post-hoc tests were performed. Pearson’s correlation coefficient was determined to assess the interrelationships between heart rate and quantitative variables. Parametric and non-parametric tests were used to assess the relationships of qualitative variables. Univariate logistic regression analysis was used to determine the risk (odds ratio) of the occurrence of cardiac arrhythmias associated with the examined factors. Calculations were made using MedCalc, version 17.2 (2017, MedCalc Software, Ostend, Belgium; https://www.medcalc.org; accessed on 24 March 2017).

## 3. Results

All groups were homogeneous in terms of age, hemoglobin, total calcium, potassium, free triiodothyronine levels, and the presence of hypertension. All groups differed significantly in terms of TSH concentration (*p* < 0.001), which was also a criterion for division into groups. The most common type of cancer was papillary thyroid carcinoma, with 41 cases (85.4%) in the full suppression group and 25 cases (92%) in the partial suppression group. Due to the small number of other types of cancer, they were grouped together. In the full suppression group, other cancer types included six cases (12.5%) of follicular carcinoma and one case (2.1%) of oncocytic carcinoma. In the partial suppression group, there were two cases (8%) of follicular cancer. The mean duration of suppressive therapy was 2 years. There were no statistically significant differences in the type of thyroid cancer or dose of levothyroxine used between study groups (Table 2).

There were no statistically significant differences in the maximum, minimum, and mean heart rates between groups. Average heart rates are presented in Table 3. The relationship between heart rate and the studied variables in all groups is shown in Table 4.

**Figure 1:**
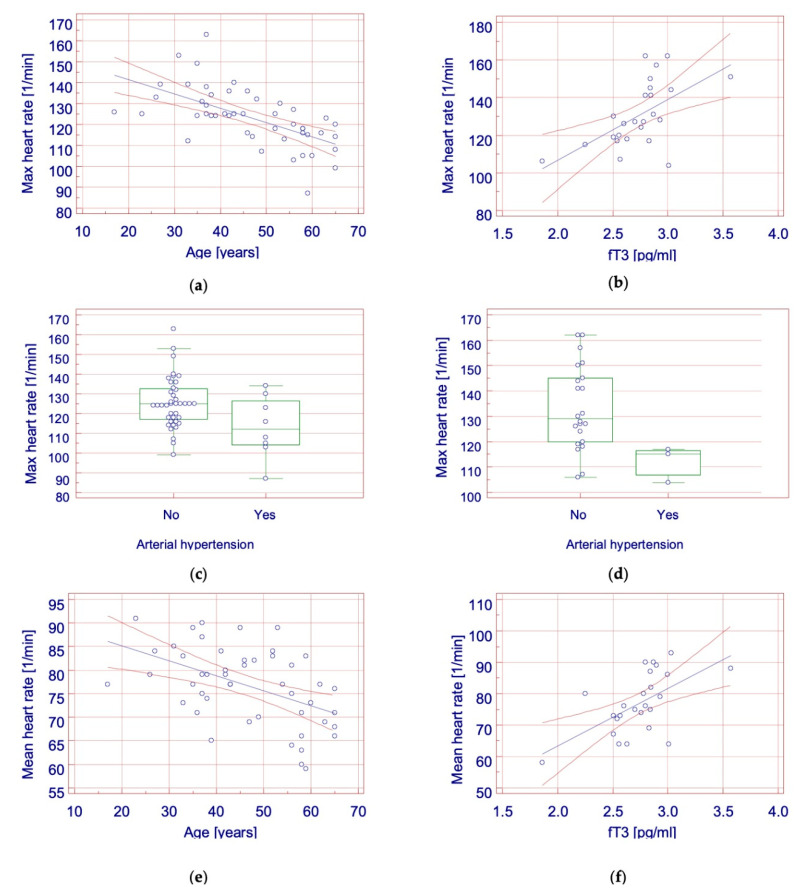
Factors affecting heart rate. (**a**) Correlation between maximal heart rate and age in group with full TSH suppression (*p* < 0.001. r = –0.609); (**b**) Correlation between maximal heart rate and free triiodothyronine serum concentration in group with full TSH suppression (*p* = 0.002. r = 0.582); (**c**) Relationship between maximal heart rate and arterial hypertension in group with full TSH suppression (*p* = 0.048); (**d**) Relationship between maximal heart rate and arterial hypertension in control group (*p* = 0.017); (**e**) Correlation between mean heart rate and age in group with full TSH suppression (r = 0.001. r = −0.486); (**f**) Correlation between mean heart rate and age in group with partial TSH suppression (r = 0.002. r = 0.582). Legend: fT3—free triiodothyronine.

There was a strong negative correlation (*p* < 0.001, r = −0.609) between age and maximum heart rate in the fully suppressed group (Figure 1a). A statistically significant positive correlation was found between maximum heart rate and the concentration of free triiodothyronine (*p* = 0.002, r = 0.582) in the control group (Figure 1b). A significant relationship was also found between the presence of hypertension and maximum heart rate in both the fully suppressed group and the control group (Figure 1c,d). In the full suppression group, the maximum heart rate in patients with arterial hypertension was lower than in patients without hypertension (113.25 ± 15.6 vs. 125.55 ± 12.818; *p* = 0.048). In the control group, the presence of hypertension was significantly associated with maximum heart rate (112 ± 7 vs. 133.318 ± 16.551; *p* = 0.017). There was a negative correlation (*p* < 0.001, r = −0.486) between age and mean heart rate in the full suppression group (Figure 1e). A significant strong positive correlation was found between mean heart rate and the concentration of free triiodothyronine (*p* = 0.002, r = 0.594) in the control group (Figure 1f). There were no significant differences between groups in the incidence of supraventricular and ventricular extrasystoles, or in the incidence of paroxysmal supraventricular tachycardia. There were no episodes of atrial fibrillation according to the adopted definition. One-way logistic regression did not show any significant factors significantly affecting arrythmias incidence in any group.

## 4. Discussion

The relationship between arrhythmias and hyperthyroidism was noticed as early as the 1940s. The first studies showed that hyperthyroidism influences the incidence of sinus tachycardia, premature supraventricular beats, and atrial fibrillation [13,14,15]. A reduced TSH concentration is a risk factor for the occurrence of atrial fibrillation [11], and subclinical hyperthyroidism has also been shown to increase the risk of this arrhythmia [16]. Along with the development of new treatment principles for differentiated thyroid cancer, the effect of exogenous subclinical hyperthyroidism caused by suppressive doses of levothyroxine on cardiac arrhythmias has emerged. The present study was conducted in the reference center for the Świętokrzyskie Voivodeship, which has the highest standardized incidence rates of thyroid cancer in the country for both women and men [4]. The large difference in the number of fully suppressed patients (*n* = 48) compared to other groups (both *n* = 25) resulted from the prospective nature of the study and more frequent use of full suppression doses in the study population. The study groups were homogeneous in terms of age, hemoglobin, total calcium, potassium, and free triiodothyronine concentrations, and the presence of arterial hypertension. There were no significant differences in the maximum, mean, and minimum heart rates between groups. Biondi et al. showed a higher mean heart rate in TSH suppressed patients in a study involving younger patients treated with higher levothyroxine doses than in our study [17]. In another study, the same authors observed that endogenous subclinical hyperthyroidism also has a significant effect on average heart rate [14]. By contrast, Shapiro et al. found no significant differences in mean heart rate between the TSH suppression group and healthy volunteers in individuals of a similar age to those in our study [18]. Ching et al. also found no differences in mean heart rate between TSH-suppressed patients and a healthy population but showed a significant effect of long-term TSH suppression on myocardial hypertrophy [19]. In a study of endogenous subclinical hyperthyroidism in patients with nodular goiter, Berghout et al. also found no significant differences in the maximum, mean, and minimum heart rates compared with controls. The authors extended the analysis to compare these parameters during sleep but did not find any significant differences [20]. In our study, we found that heart rate depended on age, the presence of arterial hypertension, and triiodothyronine level. No other studies conducted so far have analyzed the relationship between heart rate and selected clinical variables. Our study found no difference in the prevalence of supraventricular and ventricular arrhythmias between groups. The lack of significant differences in supraventricular extrasystole between TSH suppressed patients and healthy people was also shown by Shapiro et al. [18]. Patients with TSH suppression in that study had similar TSH values to those in our study, which amounted to 0.03 ± 0.04 μU/mL, whereas the daily dose of levothyroxine was higher than in our study and amounted to 192 ± 58 μg/day. Supraventricular extrasystoles (>100/24 h) were not found in any of the participants, and lower numbers of extrasystoles were found in 17.8% of fully suppressed patients versus 5.9% in the control group; however, the differences between groups were not statistically significant [18]. A significant difference between groups in terms of supraventricular extrasystole was observed by Biondi et al. [17]. By contrast, in another study on endogenous subclinical hyperthyroidism, the same authors showed no statistically significant differences in supraventricular extrasystole between individuals with subclinical hyperthyroidism and the control group. Both groups consisted of 18 people, and the adopted extrasystole standard was also 100/24 h. A significant number of supraventricular extrasystoles was found in 11% of individuals in the study group and in 6% of individuals in the control group [10]. In another study on endogenous subclinical hyperthyroidism, no significant differences were found in the presence of clinically significant supraventricular extrasystoles (>100/24 h) between the group with TSH <0.4 µU/mL and the control group, because clinically significant arrhythmia was found in 26.7% of participants in both groups [20]. Previous studies on both endogenous and exogenous subclinical hyperthyroidism have not shown statistically significant differences [10,18,20].

In the present study, short-term episodes of non-sustained paroxysmal supraventricular tachycardia were found in each group, but differences between groups were not significant. There were also no episodes of atrial fibrillation. Previous studies on factors that increase the risk of atrial fibrillation in subclinical hyperthyroidism have shown the influence of many clinical and laboratory parameters. Abonowara et al. followed up 136 patients with thyroid cancer who were taking suppressive doses of levothyroxine, assessing a routine 12 lead resting ECG for 11 years. Atrial fibrillation was found in 10.3% of patients, and in 17.5% of patients over 60 years of age [21]. In another study, Hesselink et al. compared 518 patients with differentiated thyroid cancer (TSH ≤ 0.5 µU/mL in 85.7% of participants) with a control group of 1563 people [22]. The mean age in both groups was 48.6 years, and 75% of the population were women. Atrial fibrillation was found in 42 individuals (2.7%) in the control group and in 35 individuals (6.8%) in the study group. The mean age in the study group with atrial fibrillation was much higher (59.9 ± 10.8 vs. 47.7 ± 13.9 years), and hypertension was more frequent (49% vs. 15%). Regression analysis showed a 2.5-fold increase in the risk of atrial fibrillation in patients with differentiated thyroid cancer, but no statistically significant correlation was found between TSH concentration and the occurrence of atrial fibrillation [22]. Gammage et al. analyzed 5860 patients over 64 years of age (126 with subclinical hyperthyroidism) and found that atrial fibrillation occurs almost twice as often in patients with subclinical hyperthyroidism than in euthyroid patients [23]. In a large population study involving 586,460 patients, an increased risk of atrial fibrillation was shown in patients with a decreased TSH level. In the TSH suppression group, the risk increased 1.4-fold [24]. Cappola et al. prospectively examined more than 3000 people over 64 years of age. During 13 years of follow-up, atrial fibrillation occurred in 8.5% of patients with subclinical hyperthyroidism, in 5.5% of individuals in euthyroid state, in 4.8% with subclinical hypothyroidism, and in 3.9% with hypothyroidism. Multivariate regression analysis showed that subclinical hyperthyroidism increases the risk of atrial fibrillation by two times [25]. Different results in terms of the influence of subclinical disorders of the thyroid gland on the occurrence of circulatory insufficiency were presented by Nanchen et al. after more than 3 years of observation of 5316 patients. The age range of the study population was high (70–82 years), and atrial fibrillation occurred in 9.4% of participants, with no statistically significant difference in the incidence of this arrhythmia between groups [26]. Although many studies have shown an increased risk of atrial fibrillation in patients with reduced TSH levels, no episodes of atrial fibrillation were recorded in the present study, which could be related to the lower age of the included individuals than in previous studies, a relatively low percentage of patients with diagnosed arterial hypertension, and an all-female population. The selection of the population is a limitation of the study, but it also indicates a group for which suppression therapy may be safe.

The presented study does not analyze the influence of BMI and fT4 concentration on heart rate and arrhythmias, which is a limitation in the assessment of the results. The influence of obesity on the incidence of sinus tachycardia through altered autonomic regulation of the heart rhythm has been confirmed by several studies [27,28,29,30]. Many studies have also confirmed an increased incidence of atrial fibrillation in obese subjects [31,32,33,34,35]. The influence of excess thyroid hormones on the occurrence of atrial fibrillation is well known, but a significant correlation has also been demonstrated between the fT4 concentration and the occurrence of atrial fibrillation in patients with subclinical hyperthyroidism [24]. Zhang et al. in a study of patients over 40 years of age with normal TSH levels showed a statistically significant linear relationship between the total fT4 concentration and heart rate in men, while in women it was insignificant [36].

## 5. Conclusions

(1) While maintaining normal free triiodothyronine concentrations, the use of suppressive doses of levothyroxine in patients after thyroidectomy for differentiated thyroid cancer does not cause statistically significant changes in maximum, average, or minimum heart rate.
(2) The heart rate in patients with full TSH suppression depended on age and the presence of arterial hypertension. In patients with partial TSH suppression, no correlation was found between TSH activity and heart rate. The heart rate in the control group depended on free triiodothyronine concentration and the presence of arterial hypertension.
(3) The use of suppressive doses of levothyroxine in patients after thyroidectomy for differentiated thyroid carcinoma does not significantly increase the risk of supraventricular or ventricular extrasystoles, or paroxysmal supraventricular tachycardia.
(4) The use of TSH suppression after surgical treatment of differentiated thyroid carcinomas is safe in terms of arrhythmia induction and does not provoke clinically significant arrhythmia.

## Figures and Tables

**Table 1:** Reference values in Holter electrocardiography. Modified by Kaziród-Wolski K from Interna Szczeklika 2018 (Table I.B.2-13) [7].

Arrhythmia	Age			
	16–30 Years	31–40 Years	41–60 Years	>60 Years
SVES	<50	<50	<100	<100
VES	<50	<50	<50	<100
PSVT	0	0	0	0
AF	0	0	0	0

SVES—supraventricular extrasystoles; VES—ventricular extrasystoles; PSVT—paroxysmal supraventricular tachycardia; AF—atrial fibrillation.

**Table 2:** Clinical characteristics.

Variable	Fully Suppressed TSH (*n* = 48)	Partially Suppressed TSH (*n* = 25)	Control Group (*n* = 25)	*p*-Value	Post Hoc
Age [years]	46 (37–58)	45 (40–56)	32 (26–57)	0.28	
Hemoglobin [g/dL]	13.34 (0.94)	13.26 (1.19)	13.56 (0.73)	0.50	
Total calcium [mmol/L]	2.38 (0.14)	2.36 (0.11)	2.41 (0.11)	0.37	
Potassium [mEq/L]	4.22 (0.28)	4.27 (0.32)	4.3 (0.37)	0.58	
Levothyroxine dose [μg/day]	132.14 (116–150)	121.43 (100–153)	NA	0.59	
TSH [µU/mL]	0.025 (0.0096–0.046)	0.224 (0.136–0.295)	1.121 (0.778–1.505)	<0.001	A≠B≠C
fT3 [pg/mL]	2.84 (0.38)	2.7 (0.33)	2.74 (0.31)	0.22	
Arterial hypertension					
	8 (16.7)	5 (20)	3 (12)	0.74	
Thyroid cancer					
Papillary	41 (85.4)	25 (92)	NA	0.42	
Other types	7 (14.6)	2 (8)	NA		

fT3—free triiodothyronine; NA—not applicable; TSH—thyroid stimulating hormone.

**Table 3:** Heart rates and cardiac rhythm disorders detected on Holter electrocardiography.

Variable	Fully Suppressed TSH (*n* = 48)	Partially Suppressed TSH (*n* = 25)	Control Group (*n* = 25)	*p*-Value
Heart rate [1/min]				
Maximum	123.5 (13.93)	123.48 (12.62)	130.76 (17.14)	0.10
Minimum	52 (48.5–56)	52 (47.75–57.25)	48 (44.75–55.5)	0.10
Mean	76.73 (8.12)	77.12 (6.12)	76.96 (9.56)	0.98
Arrhythmias				
SVES	6 (12.5)	4 (16)	3 (12)	0.90
VES	4 (8.3)	0 (0)	2 (8)	0.33
PSVT	4 (8.3)	3 (12)	3 (12)	0.84

SVES—supraventricular extrasystoles; VES—ventricular extrasystoles; PSVT—paroxysmal supraventricular tachycardia.

**Table 4:** Factors affecting heart rate.

Variable	Fully Suppressed (*n* = 48)		Partially Suppressed (*n* = 25)		Control Group (*n* = 25)	
Maximum heart rate						
Pearson’s correlation	*p*-Value	r	*p*-Value	r	*p*-Value	r
Age	<0.001	–0.609	0.11	–0.324	0.32	0.206
Hemoglobin	0.6	–0.078	0.85	0.040	0.81	–0.050
Total calcium	0.26	–0.167	0.78	–0.059	0.46	–0.160
Potassium	0.77	–0.044	0.43	–0.166	0.40	–0.179
Levothyroxine dose	0.62	–0.074	0.3	–0.201	Nd	Nd
TSH	0.17	0.200	0.79	–0.057	0.21	0.259
fT3	0.39	0.127	0.91	0.226	0.002	0.582
Mann-Whitney U test	*p*-Value		*p*-Value		*p*-Value	
Cancer type	0.21	NA	0.88	NA	NA	NA
Arterial hypertension	0.048	NA	0.95	NA	0.02	NA
Minimum heart rate						
Pearson’s correlation	*p*-Value	r	*p*-Value	r	*p*-Value	r
Age	0.43	–0.116	0.24	0.243	0.84	–0.043
Hemoglobin	0.85	0.029	0.94	0.015	0.95	–0.012
Total calcium	0.85	0.028	0.33	–0.204	0.78	0.059
Potassium	0.46	0.109	0.62	–0.105	0.90	–0.027
Levothyroxine dose	0.73	–0.052	0.80	–0.053	NA	NA
TSH	0.37	0.133	0.32	0.209	0.20	0.267
fT3	0.58	0.081	0.91	0.024	0.18	0.280
Mann-Whitney U test	*p*-Value		*p*-Value		*p*-Value	
Cancer type	0.14	NA	0.21	NA	NA	NA
Arterial hypertension	0.41	NA	0.97	NA	0.83	NA
Mean heart rate						
Pearson’s correlation	*p* value	r	*p* value	r	*p* value	r
Age	0.001	–0.486	0.56	–0.124	0.23	–0.247
Hemoglobin	0.71	–0.056	0.46	0.156	0.68	0.088
Total calcium	0.08	–0.256	0.31	0.211	0.97	0.009
Potassium	0.82	–0.034	0.63	–0.101	0.44	–0.165
Levothyroxine dose	0.45	–0.111	0.19	–0.273	NA	NA
TSH	0.15	0.212	0.32	0.208	0.74	0.071
fT3	0.25	0.170	0.40	0.177	0.002	0.594
Mann-Whitney U test	*p*-Value		*p*-Value		*p*-Value	
Cancer type	0.07	NA	0.88	NA	NA	NA
Arterial hypertension	0.12	NA	0.50	NA	0.3	NA

NA—not applicable; TSH—thyroid stimulating hormone; fT3—free triiodothyronine.

## Data Availability

The datasets generated during and/or analyzed during the current study are available from the corresponding author on reasonable request.
